# Assessment of the correlation between supracrestal gingival tissue dimensions and other periodontal phenotypes components via the digital registration method: a cross‑sectional study in a Chinese population

**DOI:** 10.1186/s12903-024-04158-0

**Published:** 2024-04-01

**Authors:** Kaijin Lin, Siyi Wang, Xiaofeng Xu, Lu Yu, Rui Pan, Minqian Zheng, Jin Yang, Jianbin Guo

**Affiliations:** 1 Fujian Key Laboratory of Oral Diseases & Fujian Provincial Engineering Research Center of Oral Biomaterial & Stomatological Key Lab of Fujian College and University, School and Hospital of Stomatology, Fujian Medical University, Fuzhou, 350001 China; 2 Institute of Stomatology & Research Center of Dental and Craniofacial Implants, School and Hospital of Stomatology, Fujian Medical University, Fuzhou, 350001 China; 3 Research Center of Dental and Craniofacial Implants, School and Hospital of Stomatology, Fujian Medical University, Fuzhou, 350001 China; 4 The Affiliated Hospital (Group) of Putian University, Putian, 351100 China

**Keywords:** Digital registration, Supracrestal soft tissue, Periodontal phenotype, Esthetics zone

## Abstract

**Background:**

Supracrestal gingival tissue dimensions (SGTDs) has been considered to be an essential element of periodontal phenotype (PP) components. This study aimed to explore the relationship between SGTDs and other PP components by digital superposition method that integrated cone beam computed tomography (CBCT) with intraoral scanning.

**Methods:**

This cross-sectional study was conducted at the Stomatology Hospital of Fujian Medical University. Participants were recruited based on the inclusion and exclusion criteria. The data obtained from the digital scanner (TRIOS 3, 3Shape, Denmark) and CBCT images were imported into the TRIOS software (Implant Studio, 3Shape, Denmark) for computing relevant parameters. The significant level was set at 0.05.

**Results:**

A total of 83 participants with 498 maxillary anterior teeth were finally included. The mean values of supracrestal gingival height (SGH) and the distance from the cementoenamel junction (CEJ) to the crest of the alveolar ridge (CEJ-ABC) on the buccal site were significantly higher than palatal SGH (SGH-p) and palatal CEJ-ABC (CEJ-ABC-p). Men exhibited taller CEJ-ABC and SGH-p than women. Additionally, tooth type was significantly associated with the SGH, SGH-p and CEJ-ABC-p. Taller SGH was associated with wider crown, smaller papilla height (PH), flatter gingival margin, thicker bone thickness (BT) and gingival thickness (GT) at CEJ, the alveolar bone crest (ABC), and 2 mm apical to the ABC. Smaller SGH-p displayed thicker BT and GT at CEJ, the ABC, and 2 and 4 mm apical to the ABC. Higher CEJ-ABC showed lower interproximal bone height, smaller PH, flatter gingival margin, thinner GT and BT at CEJ, and 2 mm apical to the ABC. Smaller CEJ-ABC-p displayed thicker BT at CEJ and 2 and 4 mm apical to the ABC. On the buccal, thicker GT was correlated with thicker BT at 2 and 4 mm below the ABC.

**Conclusion:**

SGTDs exhibited a correlation with other PP components, especially crown shape, gingival margin and interdental PH. The relationship between SGTDs and gingival and bone phenotypes depended on the apico-coronal level evaluated.

**Trial registration:**

This study was approved by the Biomedical Research Ethics Committee of Stomatology Hospital of Fujian Medical University (approval no. 2023-24).

**Supplementary Information:**

The online version contains supplementary material available at 10.1186/s12903-024-04158-0.

## Background

As aesthetic expectations in dentistry have risen, the criteria for aesthetics in anterior dental restorations have become more rigorous [1–4]. Achieving ideal aesthetic outcomes goes beyond merely reconstructing the crown shape and color; it necessitates a comprehensive approach to managing the surrounding soft and hard tissues. Accurate assessment of the periodontal phenotype around teeth and peri-implant is crucial for devising an appropriate treatment strategy that ensures a satisfactory esthetic outcome aligned with long-term function, comfort, and periodontal health [5–7]. Lacking a profound understanding of these periodontal features could compromise the long-term success of periodontal surgery, restorative therapy, and implant treatment [8–10].

The 2017 World Workshop on the classification of periodontal and peri-implant diseases and conditions reached a consensus that the periodontal phenotype (PP) was categorized by the gingival morphotype (GM), which includes gingival thickness (GT) and the width of keratinized tissue, and the bone morphotype (BM), namely the thickness of the buccal bone plate (BT) [11]. This consensus, however, provided limited perspectives on supracrestal gingival tissue dimensions (SGTDs), which has been recognized to be an essential element regarding PP components [12]. Clinically, SGTDs was characterized by two main measurements: the supracrestal gingival height (SGH) and the distance from the cementoenamel junction (CEJ) to the crest of the alveolar ridge (CEJ-ABC). The concept of SGH was first introduced by Smukler in 1997 [13] and was defined from a histological perspective, encompassing sulcus depth, epithelial attachment, and the connective tissue attachment of the gingiva [14]. Additionally, CEJ-ABC has been acknowledged as a pivotal indicator for evaluating alveolar bone resorption [15]. Any infringement to SGTDs might trigger inflammatory responses, which could result in the downward migration of the gingival margin and subsequent bone loss [11]. Thus, accurate assessment of SGTDs morphology is vital before aesthetic treatments, ensuring more informed clinical decisions. Previous research has highlighted considerable clinical variation in SGTDs dimensions across different dental arches, surfaces, tooth types, and nationalities [16]. However, a comprehensive study concerning SGTDs in the Chinese Han population is still lacking.

Conflicting results have emerged on the association between SGTDs and other PP [14, 17]. One study found a negative correlation between SGTDs (SGH and CEJ-ABC) and both BT and GT using intraoral clinical photographs and cone beam computed tomography (CBCT) images [12]. This finding was in accordance with Cook and collaborators [18], who employed CBCT and clinical examinations on the maxillary anterior teeth (incisors, lateral incisors, and canines) of 60 patients. They noted taller CEJ-ABC measurements in patients with a thin PP compared to those with a thick PP. In contrast, a clinical study focusing on periodontally healthy Indians conducted by Arora using transgingival probing [14], reported individuals with thicker biotypes exhibited higher SGH than those with thinner counterparts. A significant factor contributing to these varied findings seems to be the different clinical assessment methodologies used to measure SGTDs and other PP [16].

Various methods for measuring PP components have been documented, encompassing histological measurements, clinical examinations [19] and digital assessment [20, 21]. Previous studies have demonstrated that digital assessment using CBCT imaging was effective and noninvasive to characterize the phenotypic features of the periodontium compared with clinical methods and histologic assessments [22–26]. In comparison to CBCT, intraoral scanning yield greater precision in capturing soft tissue morphotypes, encompassing aspects such as crown and GM, the curve of the gingival margin, and papilla height (PH) [27, 28]. Research has suggested that integrating CBCT images with intraoral scanning data could comprehensively assess the periodontal phenotype of both bone and soft tissue in clinical practice [21, 29, 30]. To date, there is a limited number of studies that have utilized this multidimensional methodology to investigate the correlation between SGTDs and other PP. Therefore, the aim of this cross-sectional study was to assess the relationship between SGTDs (SGH and CEJ-ABC) and crown morphology (CM), GM and BM on both buccal and palatal sides, with the digital approach involving CBCT images and intraoral scanning. The null hypothesis was that there was no significant correlation between SGTDs (SGH and CEJ-ABC) and other PP of periodontally healthy Han nationality youth in the maxillary anterior zone.

## Methods

### Ethical considerations

This study was approved by the Biomedical Research Ethics Committee of Stomatology Hospital of Fujian Medical University, China (approval no. 2023-24). Each participant provided informed consent after receiving a comprehensive explanation regarding the nature, risks, and benefits of this clinical investigation [31].

### Study design and population

This cross-sectional study was conducted at the Department of Oral and Maxillofacial Surgery, Affiliated Stomatological Hospital of Fujian Medical University (China) from November 2022 to June 2023. This study was carried out in accordance with the guideline for the Strengthening the Reporting of Observational Studies in Epidemiology (STROBE) Statement [32].

Inclusion criteria were: (1) Han nationality; (2) Age range of 18–25 years; (3) Healthy periodontal status: gingival index of 0, no bleeding upon probing, probing depth up to 3 mm, absence of gingival recession, and no attachment loss; (4) Complete natural maxillary dentition without any fillings, prosthetic crowns, endodontic treatments, decay, root resorption, or any misalignment in the teeth under examination; (5) No crowded dentition in the maxillary anterior region. Exclusion criteria were: (1) Prior periodontal surgery in the maxillary anterior region; (2) Intake of any medication influencing bone or soft tissue metabolism; (3) Previous orthodontic treatments; (4) Smoking; (5) Withdrawal of consent.

### Data acquisition

Before the test, each participant received instructions on oral hygiene maintenance. Additionally, they underwent professional mechanical plaque removal a week prior to the assessment. The intraoral scanner, TRIOS 3 (3Shape, Copenhagen, Denmark), was utilized following a standardized scan protocol recommended by a prior study [33]. Prior to scanning, care was taken to thoroughly dry the participants’ dental surfaces and soft tissues. All the intraoral scans were saved in polygon (PLY) format. The collection of CBCT images adhered to a standardized procedure in line with manufacturer guidelines and followed the principle of as low as diagnostically acceptable (ALADA) according to the patient’s needs [34]. Before using the CBCT machine (KaVo-i-CAT 17–19, KaVo, Germany), participants were equipped with lead aprons and positioned in an intercuspal position [35]. The scan had settings of 220 V, 50 Hz, 1150 VA, with a field of 16 cm x 8 cm, voxel size at 0.2 mm, and an exposure time of 26.9 s. CBCT scans were saved as digital imaging and communication in medicine (DICOM) files. Subsequently, Mimics Medical 20.0 (Materialise, Belgium) was utilized for image processing and maxillary bone mathematical analysis [36], which allowed for automatic superimposition of images. Both the PLY and DICOM files were imported into the TRIOS software (Implant Studio, 3Shape, Denmark) and underwent standardized alignment. The initial registration of the CBCT and intraoral scans was performed using automatic registration in the software, followed by manual adjustments for fine-tuning. After completing the registration, the alignment accuracy was observed.

### Clinical parameters and measurement

**Fig. 1:**
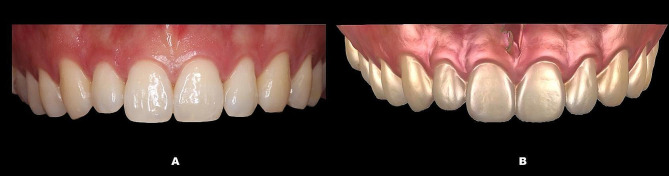
Intra-oral image (**A**) digital models (**B**)

**Fig. 2:**
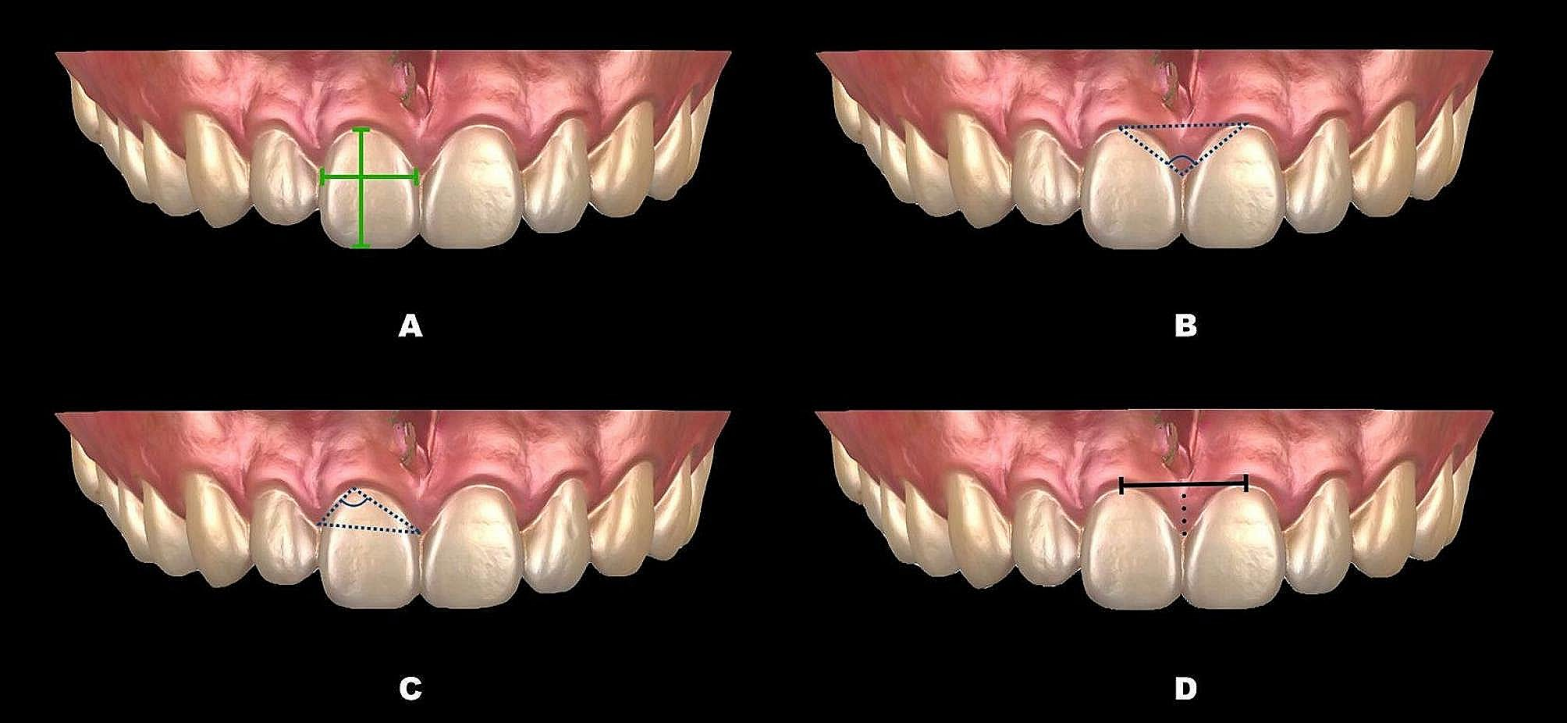
The anterior maxillary teeth measured are represented in the following figures: crown length (vertical green line), crown width (horizontal green line) (**A**); papilla angle (the angle between the zenith of the gingival papilla and the gingival zeniths of the corresponding adjacent teeth) (**B**); gingival angle (the angle between the gingival zenith and the zeniths of the corresponding adjacent gingival papillae) (**C**); papilla width (horizontal black line), papilla height (vertical dotted line) (**D**)

**Fig. 3:**
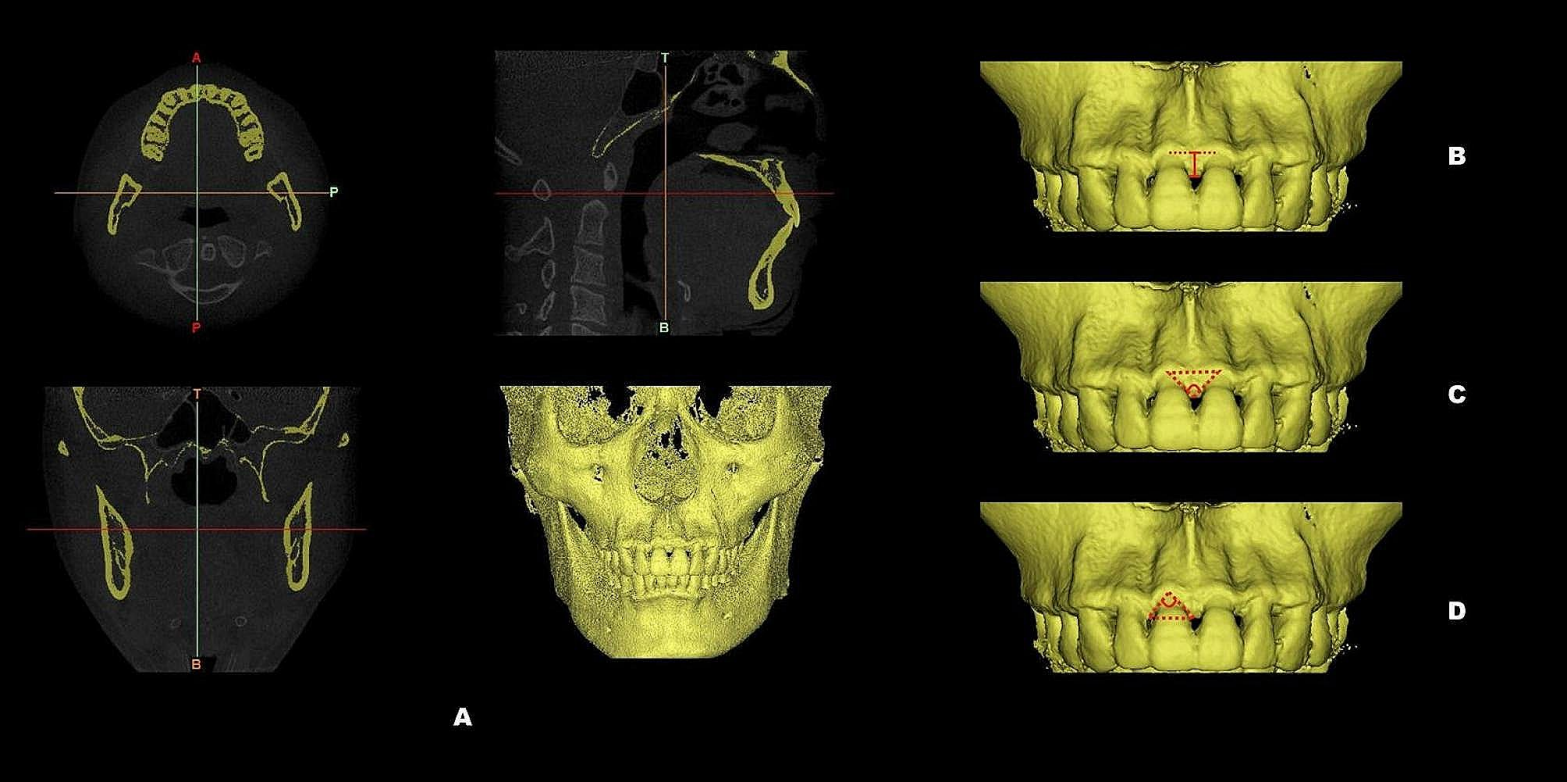
The DICOM files were imported into Mimics Medical 20.0 (Materialise, Belgium) to reconstruct the maxillary bone model (**A**); Interproximal bone height (IBH, vertical red line) (**B**); Interproximal bone angle (IBA, the angle between the zenith of the interproximal bone and the corresponding adjacent bone margin zeniths) (**C**); Bone margin angle (BMA, The angle between the bone margin zenith and the zeniths of the corresponding adjacent interproximal bone) (**D**)

**Fig. 4:**
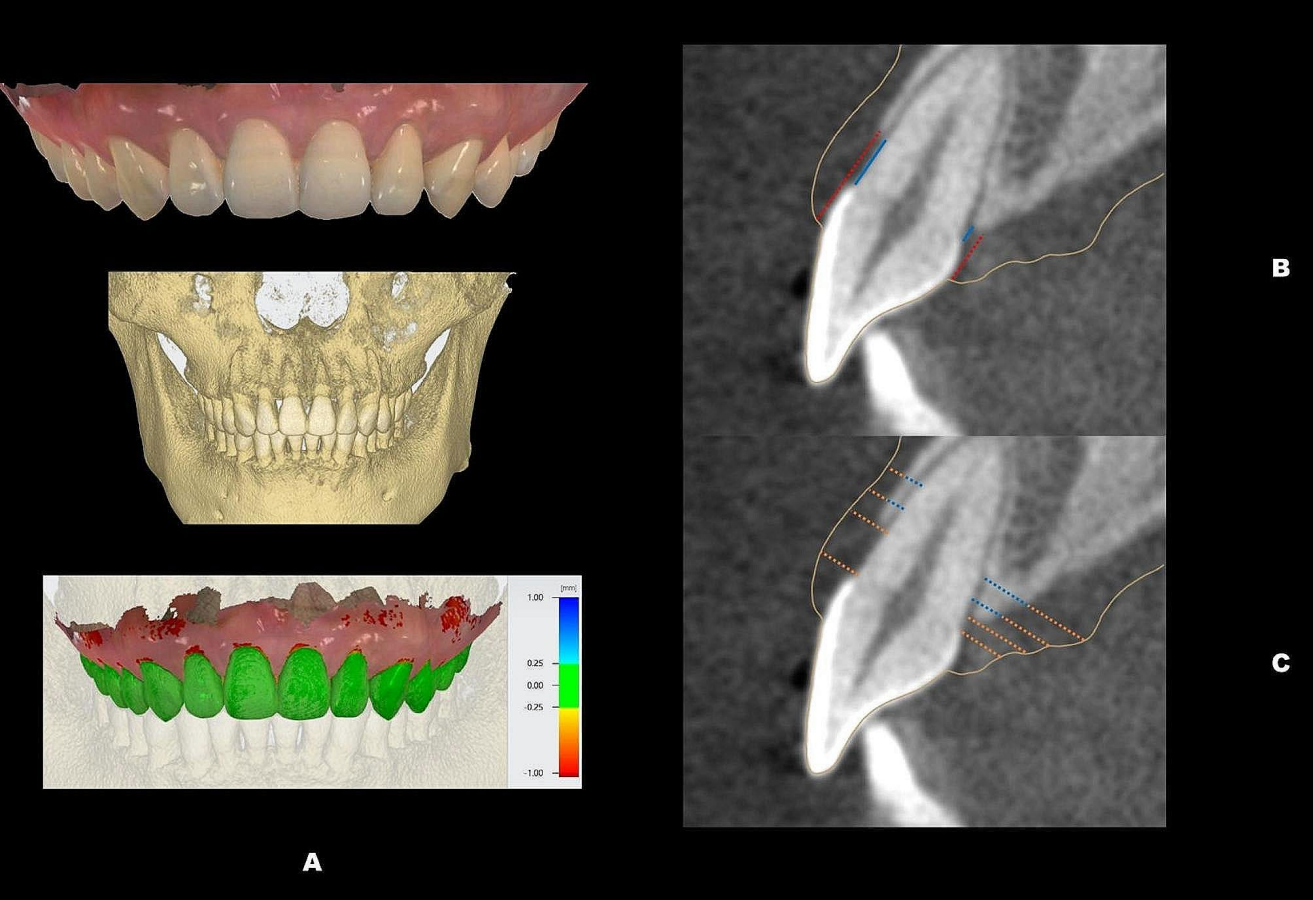
Both PLY and DICOM files were imported into the TRIOS software (Implant Studio, 3Shape, Copenhagen, Denmark) (**A**); The supracrestal gingival height (red dotted line) and the distance from the cementoenamel junction to the bone crest (blue line) (**B**); The facial and palatal gingival thickness at the levels of the cementoenamel junction, the bone crest, 2 and 4 mm apical to the bone crest (orange dotted line) and the facial and palatal bone plate thickness at the levels of 2 and 4 mm apical to the bone crest (blue dotted line) (**C**)

A calibrated dentist (Kaijin Lin) carried out all the measurements, and to ensure accuracy, these measurements were retaken after a one-week interval. All intraoral scans were imported into Geomagic Control X (3D Systems, Rock Hill, SC) to generate digital models and compute the associated indices. (Fig. 1) Clinical investigative parameters were assessed: crown length (CL), crown width (CW), crown shape (CW/CL), gingival angle (GA), papilla angle (PA), papilla width (PW), and PH. (Table 1; Fig. 2) Parameters associated with BM using CBCT images included bone margin angle (BMA), interproximal bone angle (IBA), and interproximal bone height (IBH). (Table 1; Fig. 3) When integrating data from CBCT images with intraoral scanning, the following relevant parameters were measured: facial SGH (SGH), palatal SGH (SGH-p), facial distance from CEJ to the crest of alveolar ridge (CEJ-ABC), palatal CEJ-ABC (CEJ-ABC-p), facial GT at the CEJ (GT_cej_), palatal GT at the CEJ (GT_cej_-p), facial GT at the alveolar bone crest (ABC) (GT_abc_), palatal GT at the ABC (GT_abc_-p), facial GT at 2 mm apical to the ABC (GT_1_), palatal GT at 2 mm apical to the ABC (GT_1_-p), facial GT at 4 mm apical to the ABC (GT_2_), palatal GT at 4 mm apical to the ABC (GT_2_-p), facial BT at 2 mm apical to the ABC (BT_1_), palatal BT at 2 mm apical to the ABC (BT_1_-p), facial BT at 4 mm apical to the ABC (BT_2_) and palatal BT at 4 mm apical to the ABC (BT_2_-p). (Table 1; (Fig. 4A) (Fig. 4B and C)

**Table 1:** The definition, measurement methods and classification of different periodontal features

Classification	Parameters	Method	Definition
CM	CL	IOS	The shortest distance from the gingival zenith to the incisal edge
	CW		The distance between the proximal tooth surfaces measured at the border between the middle and cervical portions
	Crown shape		CW/CL
GM	GA	IOS	The angle between the gingival zenith and the zeniths of the corresponding adjacent gingival papillae
	PA		The angle between the zenith of the gingival papilla and the gingival zeniths of the corresponding adjacent teeth
	PW		The distance between the gingival zeniths of the adjacent teeth
	PH		The shortest distance from the zenith of the papilla to a line connects the gingival zeniths of the adjacent teeth
	SGH	IOS + CBCT	The distance from the gingival margin to the ABC
	CEJ-ABC		The distance between the CEJ and the ABC
	GT		Gingival thickness, comprise GT_cej_, GT_abc_, GT_1_ and GT_2_
	GT-p		GT on the palatal side, comprise GT_cej_-p, GT_abc_-p, GT_1_-p and GT_2_-p
BM	BMA	CBCT	The angle between the bone margin zenith and the zeniths of the corresponding adjacent interproximal bone
	IBA		The angle between the zenith of the interproximal bone and the corresponding adjacent bone margin zeniths
	IBH		The shortest distance from the zenith of the interproximal bone to a line connects the adjacent bone margin zeniths
	BT	IOS + CBCT	Bone thickness, comprise BT_1_ and BT_2_
	BT-p		Bone thickness on the palatal side, comprise BT_1_-p and BT_2_-p

CM: crown morphology; GM: gingival morphology; BM: bone morphology; IOS: intraoral scanning; CBCT: cone beam computed tomography; ABC: the crest of alveolar ridge; GT_cej_: gingival thickness (GT) at the level of the cementoenamel junction (CEJ); GT_abc_: GT at the ABC; GT_1_: GT from 2 mm apical to the ABC; GT_2_: GT from 4 mm apical to the ABC; GT_cej_-p: GT at the level of the CEJ on the palatal side; GT_abc_-p: GT at the ABC on the palatal side; GT_1_-p: GT from 2 mm apical to the ABC on the palatal side; GT_2_-p: GT from 4 mm apical to the bone crest on the palatal side; BT_1_: BT at 2 mm apical to the ABC; BT_2_: BT at 2 mm apical to the ABC; BT_1_-p: BT at 2 mm apical to the ABC on the palatal side; BT_2_-p: BT at 2 mm apical to the ABC on the palatal side

### Statistical analysis

All data were analyzed using SPSS 22.0 statistical software (SPSS version 22.0, Chicago, IL). The reliability analysis of all variables was conducted using the intraclass correlation coefficient (ICC). The Shapiro-Wilk test and Probability-probability Plot were employed to assess the Gaussian distribution of all variables. Data following a normal distribution is presented as mean ± standard deviation ($\overline{x}$ ± s). Paired sample tests were conducted to determine if there was a statistical difference between the data from the left and right sides. If no statistical difference was found, the data for corresponding teeth on both sides were merged. The comparison of continuous variables at different tooth positions used Student’s t-test and one-way analysis of variance (ANOVA). Various variables’ correlations were determined using Pearson correlations. The Pearson r score was adopted (*r* = 0.1–0.3, weak correlation; *r* = 0.4–0.6; moderate correlation; *r* > 0.7, strong correlation) [37]. Statistical significance was set at 0.05.

### Sample size calculation

In this research, we adopted a cross-sectional study design, analyzing at the level of individual teeth. Based on relevant literature [14, 17] and preliminary trial results, the minimum sample size was determined using PASS software (PASS 2020, Kaysville, American). It was estimated at 424 teeth, accounting for a sampling error of α = 0.05, a study power of 0.9, and an anticipated dropout rate of 20%.

## Results

Of the total 134 participants included in the study after initial screening. Eight individuals were excluded due to a history of smoking, previous orthodontic treatment, or root canal treatment. Then 43 subjects were excluded because of crowding or misalignment of the maxillary anterior teeth. Therefore, a total of 498 anterior teeth from 83 participants (40 males and 43 females), with an average age of 21.6 ± 2.8 years old, were finally included in this study. ICC analysis indicated a high level of reliability in digital assessments (κ score = 0.927), CBCT assessments (κ score = 0.935) and combination methods (κ score = 0.902).

### Mean SGH and CEJ-ABC at different genders and tooth types

The mean values of SGH (3.29 ± 0.61 mm) and CEJ-ABC (1.46 ± 0.47 mm) on the buccal site were significantly higher than palatal SGH-p (2.91 ± 0.51 mm) and CEJ-ABC-p (0.98 ± 0.31 mm) (*P* < 0.01). There was a significant gender difference in CEJ-ABC and SGH-p measurements. For CEJ-ABC, male participants averaged 1.56 mm, while female participants averaged 1.37 mm (*P* < 0.05). In terms of SGH-p, males recorded an average of 3.02 mm, whereas females averaged 2.83 mm (*P* < 0.01). Additionally, tooth type was significantly associated with the SGH, SGH-p and CEJ-ABC-p (*P* < 0.01). Central incisors performed the highest SGH (3.46 ± 0.58 mm), followed by lateral incisors (3.39 ± 0.62 mm) and canines (3.03 ± 0.54 mm). Regarding SGH-p, canines showed a higher SGH-p (3.21 ± 0.54 mm) than lateral incisors (2.88 ± 0.46 mm) and central incisors (2.65 ± 0.38 mm). Canines exhibited the highest CEJ-ABC-p (1.06 ± 0.30 mm) compared to incisors (0.96 ± 0.31 mm) and lateral incisors (0.92 ± 0.31 mm). (Table 2)

**Table 2:** Periodontal variables at different genders and teeth

	Overall				Central incisors				Lateral incisors				Canines		
	Mean ± SD	Men Mean ± SD	Women Mean ± SD		Mean ± SD	Men Mean ± SD	Women Mean ± SD		Mean ± SD	Men Mean ± SD	Women Mean ± SD		Mean ± SD	Men Mean ± SD	Women Mean ± SD
**CL(mm)**	9.02 ± 1.20	9.52 ± 1.11	8.62 ± 1.12^**^		9.69 ± 1.00^§§^^^	10.17 ± 0.78	9.30 ± 1.00^**^		8.28 ± 1.05^##^^^	8.82 ± 0.98	7.85 ± 0.90^**^		9.08 ± 1.12^##§§^	9.57 ± 1.13	8.69 ± 0.95^**^
**CW(mm)**	6.71 ± 0.82	6.92 ± 0.76	6.54 ± 0.83^**^		7.43 ± 0.48^§§^^^	7.58 ± 0.43	7.30 ± 0.49^*^		5.86 ± 0.59^##^^^	6.11 ± 0.54	5.67 ± 0.57^*^		6.84 ± 0.43^##§§^	7.06 ± 0.39	6.66 ± 0.38^**^
**CW/CL**	0.75 ± 0.09	0.73 ± 0.08	0.76 ± 0.09^*^		0.77 ± 0.08^§§^	0.75 ± 0.07	0.79 ± 0.08^*^		0.71 ± 0.08^##^^^	0.70 ± 0.08	0.73 ± 0.08^*^		0.76 ± 0.09^§§^	0.75 ± 0.09	0.77 ± 0.08
**GA(°)**	94.61 ± 10.38	92.29 ± 10.07	96.46 ± 10.30^*^		97.03 ± 9.36^^^^	94.77 ± 9.10	98.84 ± 9.28		96.47 ± 9.82^^^^	93.16 ± 9.45	99.11 ± 9.41		90.32 ± 10.69^##§§^	88.94 ± 10.96	91.42 ± 10.48
**PA(°)**	102.21 ± 11.35	99.40 ± 10.70	104.46 ± 11.40^**^		103.93 ± 11.59^§^	100.59 ± 10.84	106.60 ± 11.61^*^		100.00 ± 10.12^#^	96.33 ± 8.25	102.93 ± 10.60^*^		102.71 ± 12.04	101.28 ± 12.26	103.85 ± 11.90
**PW(mm)**	8.66 ± 0.92	8.84 ± 0.80	8.51 ± 0.97^**^		9.39 ± 0.77^§§^^^	9.46 ± 0.63	9.34 ± 0.88		8.30 ± 0.59^##^	8.52 ± 0.66	8.13 ± 0.47		8.28 ± 0.88^##^	8.53 ± 0.75	8.07 ± 0.93^*^
**PH(mm)**	3.55 ± 0.78	3.84 ± 0.76	3.31 ± 0.72^**^		3.82 ± 0.81^§§^^^	4.14 ± 0.71	3.56 ± 0.80^**^		3.47 ± 0.64^##^	3.79 ± 0.59	3.22 ± 0.56^**^		3.37 ± 0.81^##§§^	3.58 ± 0.87	3.19 ± 0.73^*^
**IBH(mm)**	2.63 ± 0.48	2.78 ± 0.51	2.50 ± 0.42^**^		2.82 ± 0.55^§§^^^	2.98 ± 0.63	2.70 ± 0.43^*^		2.58 ± 0.39^##^	2.71 ± 0.40	2.48 ± 0.36^*^		2.48 ± 0.43^##^	2.66 ± 0.43	2.33 ± 0.38^**^
**BMA(°)**	112.70 ± 9.66	110.98 ± 10.03	114.08 ± 9.17^*^		113.36 ± 8.38	112.88 ± 9.24	113.74 ± 7.73		112.57 ± 10.82	109.02 ± 10.17	115.41 ± 10.59		112.17 ± 9.74	111.03 ± 10.59	113.09 ± 9.03
**IBA(°)**	115.43 ± 10.08	112.83 ± 9.85	117.50 ± 9.81^**^		114.28 ± 10.78^^^	111.18 ± 11.12	116.76 ± 9.95^*^		113.62 ± 9.82^^^^	110.59 ± 8.07	116.05 ± 10.49^*^		118.38 ± 9.02^#§§^	116.72 ± 9.20	2.33 ± 0.38
**SGH(mm)**	3.29 ± 0.61	3.26 ± 0.63	3.31 ± 0.59		3.46 ± 0.58^^^^	3.43 ± 0.62	3.47 ± 0.53		3.39 ± 0.62^^^^	3.29 ± 0.63	3.47 ± 0.61		3.03 ± 0.54^##§§^v	3.06 ± 0.57	3.00 ± 0.52
**CEJ-ABC(mm)**	1.46 ± 0.47	1.56 ± 0.53	1.37 ± 0.40^*^		1.47 ± 0.54	1.66 ± 0.62	1.32 ± 0.43^**^		1.46 ± 0.42	1.56 ± 0.44	1.37 ± 0.40^**^		1.44 ± 0.44	1.47 ± 0.51	1.42 ± 0.39
**GT**_**cej**_	1.20 ± 0.32	1.21 ± 0.38	1.18 ± 0.26		1.44 ± 0.34^§§^^^	1.47 ± 0.45	1.41 ± 0.21		1.09 ± 0.23^##^	1.08 ± 0.27	1.10 ± 0.21		1.06 ± 0.22^##^	1.08 ± 0.25	1.04 ± 0.20
**GT**_**abc**_	1.42 ± 0.31	1.45 ± 0.34	1.41 ± 0.28		1.70 ± 0.27^§§^^^	1.77 ± 0.29	1.64 ± 0.24^*^		1.28 ± 0.24^##^	1.27 ± 0.24	1.29 ± 0.23^*^		1.30 ± 0.22^##^	1.30 ± 0.23	1.29 ± 0.22
**GT**_**1**_	0.67 ± 0.25	0.69 ± 0.30	0.65 ± 0.20^**^		0.65 ± 0.30	0.70 ± 0.39	0.61 ± 0.21		0.69 ± 0.23	0.68 ± 0.27	0.70 ± 0.21		0.66 ± 0.22	0.68 ± 0.25	0.64 ± 0.19
**GT**_**2**_	0.67 ± 0.23	0.72 ± 0.23	0.63 ± 0.23^**^		0.66 ± 0.18^§^	0.72 ± 0.17	0.62 ± 0.18^*^		0.75 ± 0.23^#^^^	0.80 ± 0.20	0.70 ± 0.25^*^		0.60 ± 0.26^§§^	0.63 ± 0.27	0.57 ± 0.25
**BT**_**1**_	0.90 ± 0.26	0.92 ± 0.32	0.88 ± 0.20		0.95 ± 0.32^^^	0.99 ± 0.42	0.91 ± 0.21		0.89 ± 0.23	0.88 ± 0.27	0.90 ± 0.21		0.86 ± 0.22^#^	0.88 ± 0.25	0.84 ± 0.20
**BT**_**2**_	0.74 ± 0.25	0.79 ± 0.25	0.71 ± 0.25^*^		0.82 ± 0.18^§§^	0.84 ± 0.15	0.80 ± 0.20		0.59 ± 0.17^##^^^	0.64 ± 0.19	0.55 ± 0.15		0.82 ± 0.31^§§^	0.89 ± 0.31	0.76 ± 0.31
**SGH-p**	2.91 ± 0.51	3.02 ± 0.51	2.83 ± 0.49^**^		2.65 ± 0.38^§§^^^	2.68 ± 0.38	2.63 ± 0.39		2.88 ± 0.46^##^^^	3.01 ± 0.46	2.77 ± 0.43		3.21 ± 0.54^##§§^	3.36 ± 0.45	3.09 ± 0.53^*^
**CEJ-ABC-p**	0.98 ± 0.31	1.00 ± 0.33	0.96 ± 0.30		0.96 ± 0.31	0.98 ± 0.31	0.95 ± 0.31		0.92 ± 0.31^^^^	0.91 ± 0.28	0.93 ± 0.34		1.06 ± 0.30^§§^	1.47 ± 0.51	1.01 ± 0.24
**GT**_**cej**_ **-p**	1.88 ± 0.51	1.92 ± 0.49	1.85 ± 0.52		1.71 ± 0.40^§^^^	1.76 ± 0.46	1.67 ± 0.35		1.88 ± 0.62^#^^	1.96 ± 0.60	1.81 ± 0.64		2.06 ± 0.42^##§^	1.08 ± 0.25	2.07 ± 0.46
**GT**_**abc**_ **-p**	2.56 ± 0.59	2.67 ± 0.54	2.48 ± 0.61^*^		2.41 ± 0.50^^^^	2.51 ± 0.51	2.33 ± 0.49		2.56 ± 0.70	2.75 ± 0.68	2.42 ± 0.69		2.72 ± 0.50^##^	1.30 ± 0.25	2.70 ± 0.60
**GT**_**1**_ **-p**	2.77 ± 0.58	2.90 ± 0.52	2.66 ± 0.60^**^		2.40 ± 0.49^§§^^^	2.53 ± 0.47	2.30 ± 0.48^*^		2.84 ± 0.50^##^^	3.06 ± 0.44	2.68 ± 0.48^*^		3.05 ± 0.56^##§^	0.68 ± 0.25	3.00 ± 0.63
**GT**_**2**_ **-p**	2.96 ± 0.63	3.03 ± 0.59	2.91 ± 0.66		2.54 ± 0.50^§§^^^	2.57 ± 0.49	2.52 ± 0.51		2.84 ± 0.55^##^^^	2.96 ± 0.39	2.75 ± 0.64		3.51 ± 0.40^##§§^	0.63 ± 0.27	3.46 ± 0.42
**BT**_**1**_ **-p**	1.40 ± 0.39	1.47 ± 0.38	1.35 ± 0.39^*^		1.53 ± 0.52^§§^^^	1.69 ± 0.40	1.41 ± 0.41^**^		1.32 ± 0.33^##^	1.38 ± 0.33	1.27 ± 0.33^**^		1.36 ± 0.37^##^	0.88 ± 0.25	1.36 ± 0.42
**BT**_**2**_ **-p**	2.29 ± 0.72	2.50 ± 0.73	2.11 ± 0.67^**^		2.42 ± 0.74^§^	2.79 ± 0.76	2.12 ± 0.58^**^		2.12 ± 0.71^##^	2.39 ± 0.69	1.90 ± 0.66^**^		2.33 ± 0.69	0.89 ± 0.31	2.32 ± 0.71

*significantly different compared to males’ group (*:*P* < 0.05, **: *P* < 0.01)

#significantly different compared to central incisors group (#:*P* < 0.05, ##: *P* < 0.01)

**§**significantly different compared to lateral incisors group (**§**: *P* < 0.05, **§§**: *P* < 0.01)

^significantly different compared to canines group (^:*P* < 0.05,^^: *P* < 0.01)

### Correlation analysis between SGTDs (SGH and CEJ-ABC) and other PP

No statistical difference in each PP variable was observed between contralateral teeth with the same name (*P* > 0.05). Therefore, the data for contralateral teeth with the same name were combined. The relationship between SGH and CEJ-ABC and other periodontal features is shown in Table 2. SGH and CEJ-ABC show a positive correlation, both in the buccal (*r* = 0.253, *P* < 0.01) and palatal site (*r* = 0.220, *P* < 0.01).

For SGH, on the buccal side, it exhibited a positive correlation with crown shape (CW/CL) (*r* = 0.299, *P* < 0.001), GA (*r* = 0.467, *P* < 0.001), PA (*r* = 0.385, *P* < 0.001), GT_cej_ (*r* = 0.427, *P* < 0.001), GT_abc_ (*r* = 0.283, *P* < 0.001), GT_1_ (*r* = 0.338, *P* < 0.001) and BT_1_(*r* = 0.413, *P* < 0.001), while there was a negative correlation with CL (*r* = -0.292, *P* < 0.001) and PH (*r* = -0.317, *P* < 0.001). On the palatal site, SGH-p exhibited a positive correlation with GT_abc_ (*r* = 0.399, *P* < 0.001), GT_cej_ (*r* = 0.464, *P* < 0.001), GT_1_(*r* = 0.511, *P* < 0.001), GT_2_(*r* = 0.474, *P* < 0.001). (Table 3)

**Table 3:** Correlation between the periodontal supracrestal soft tissue dimensions and other periodontal features

			Crown morphology			Gingival morphology				Bone morphology			Gingival morphology				Bone morphology	
Site			CL	CW	CW/CL	GA	PA	PW	PH	BMA	IBA	IBH	GT_cej_/ GT_cej_-p	GT_abc_/ GT_abc_-p	GT_1_/GT_1_-P	GT_2_/ GT_2_-p	BT_1_/ BT_1_-p	BT_2_/ BT_2_-p
Facial	SGH	Pearson r	-0.292*	-0.025	0.299*	0.467*	0.385*	0.080	-0.317*	-0.057	-0.120	0.123	0.427*	0.283*	0.388*	-0.063	0.413*	-0.028
		p-value	0.000	0.715	0.000	0.000	0.000	0.241	0.000	0.403	0.077	0.071	0.000	0.000	0.000	0.354	0.000	0.678
	CEJ-ABC	Pearson r	0.278*	0.080	-0.234*	-0.211*	-0.203*	0.63	0.222*	-0.148*	-0.181*	0.173*	-0.201*	-0.083	-0.217*	-0.061	-0.230*	-0.003
		p-value	0.000	0.241	0.001	0.002	0.003	0.354	0.001	0.029	0.008	0.011	0.003	0.223	0.001	0.372	0.001	0.964
Palatal	SGH-p	Pearson r	-0.101	-0.130	-0.027	-0.016	0.120	-0.245*	-0.254*	-0.060	0.069	-0.142*	0.464*	0.399*	0.511*	0.474*	-0.018	0.013
		p-value	0.141	0.057	0.692	0.818	0.079	0.000	0.000	0.383	0.312	0.038	0.000	0.000	0.000	0.000	0.787	0.853
	CEJ-ABC-p	Pearson r	0.073	0.021	-0.060	-0.035	0.058	-0.007	-0.020	-0.014	-0.024	-0.013	-0.206*	-0.060	0.071	0.212*	-0.150*	-0.174*
		p-value	0.287	0.755	0.383	0.604	0.396	0.920	0.774	0.837	0.726	0.854	0.002	0.383	0.297	0.002	0.028	0.011

The facial GTs and BTs across all levels are exclusively compared with SGH and CEJ-ABC. The palatal GTs and BTs across all levels are exclusively compared with SGH-p and CEJ-ABC-p

For CEJ-ABC, on the buccal site, it exhibited a positive correlation with CL (*r* = 0.278, *P* < 0.001), PH (*r* = 0.222, *P* < 0.001) and IBH (*r* = 0.173, *P* < 0.001), while it showed a negative correlation with CW/CL (*r* = -0.234, *P* < 0.001), GA (*r* = -0.211, *P* < 0.001), PA (*r* = -0.223, *P* < 0.01), BMA (*r* = -0.148, *P* < 0.05), IBA (*r* = -0.181, *P* < 0.01), GT_cej_ (*r* = -0.201, *P* < 0.01), GT_1_ (*r* = -0.217, *P* < 0.001), BT_1_ (*r* = -0.230, *P* < 0.001).On the palatal side, CEJ-ABC-p exhibits a negative correlation with GT_cej_ (*r* = -0.206, *P* < 0.01), BT_1_ (*r* = -0.150, *P* < 0.05), BT_2_ (*r* = -0.174, *P* < 0.05). (Table 3)

### Correlation analysis between GM and BM

The relationship between gingival morphology and bone morphology is shown in Table 4. GA exhibited a significant positive correlation with BMA (*r* = 0.315, *P* < 0.001). PA was positively correlated with the IBA (*r* = 0.332, *P* < 0.001). A positive correlation was detected between PH and IBH (*r* = 0.321, *P* < 0.001).

**Table 4:** Correlation between the gingival and bone morphology

Gingival morphology	Bone morphology		
GA	BMA	Pearson r	0.315*
		p-value	0.000
PA	IBA	Pearson r	0.332*
		p-value	0.000
PH	IBH	Pearson r	0.321*
		p-value	0.000
GT_1_	BT_1_	Pearson r	0.463*
		p-value	0.000
GT_2_	BT_2_	Pearson r	0.181*
		p-value	0.008
GT_1_-p	BT_1_-p	Pearson r	0.131
		p-value	0.055
GT_2_-p	BT_2_-p	Pearson r	0.027
		p-value	0.688

The gingival angle (GA) corresponds to the bone margin angle (BMA), the papilla angle (GA) corresponds to the underlying interproximal bone angle (IBA), and the papilla height (PH) corresponds to the underlying interproximal bone height (IBH). GT_1_ and BT_1_ refer to the gingival thickness and alveolar bone thickness 2 mm below the crest of the alveolar ridge, while GT_2_ and BT_2_ refer to the gingival thickness and alveolar bone thickness 4 mm below the crest of the alveolar ridge

The correlation analysis revealed that at 2 and 4 mm below the ABC on the buccal aspect, the GT is positively correlated with BT (*r* = 0.463 and 0.181, *P* < 0.001). However, no significant correlation was found between GT and BT on the palatal aspect(*P* > 0.05).

## Discussion

In this study, we observed a significant correlation between SGTDs and CM (CW/CL, CL), GM (GA, PA, GT) and BM (BT) in the anterior maxillary region. Therefore, the null hypothesis was partially rejected. To the best of our knowledge, this is the most comprehensive cross-sectional investigation utilizing CBCT and intraoral scanning to explore the relationship between SGTDs and other periodontal characteristics. Meanwhile, we introduced several angular metrics (IBA, BMA and PA) that captured the curvature of the gingival papillae and interdental bone crest for the first time, serving as an enhancement to the characterization of GM and BM. This study adopted a digital multidimensional methodology superimposing CBCT imaging and digital intraoral scan files. This allowed for the simultaneous measurement of both soft (GT) and hard (BT) tissues at consistent levels across varying depths [5].

This study revealed that among the 83 participants included in this study, SGH on the facial side (3.29 mm) was consistent with the mean value reported by Couso-Queiruga et al. (3.26 mm) [12], who evaluated 587 maxillary anterior teeth (central incisor, lateral incisors, and canines) from 87 periodontally healthy Brazilians. However, this deviated slightly from measurements by the study conducted by Fischer (2.63 mm) [17], which assessed 80 Germans by observing gingiva transparency, and Arora (3.5 mm) [14], which measured a total of 23 individuals (14 males and 9 females) and 1932 sites (central incisor, premolar and molar) by the visibility of periodontal probe. The discrepancy could be attributed to the measurement methods used and the populations being included. Additionally, we observed an average facial CEJ-ABC measurement of 1.46 ± 0.47 mm, which is notably smaller than findings from previous research. Silva et al. found that the CEJ-ABC in the maxillary anterior region averaged 2.92 mm with the use of CBCT [24]. Similarly, Nahass reported CEJ-ABC measurements of 2.10 mm for the maxillary central incisor and 2.09 mm for the lateral incisor [38]. One potential reason for this discrepancy might be our inclusion of volunteers with healthy periodontal conditions, which result in a lower level of bone resorption being observed.

A statistically positive correlation was detected between SGH and the crown shape, gingival margin, and interdental PH. At CEJ, ABC and 2 mm apical to the ABC, taller SGH exhibited thicker GT and BT. However, at the site 4 mm apical to the ABC, no significant relationship between SGH and GT and BT was observed. Rodrigues and colleagues reported findings in alignment with ours, suggesting that the determination of thin and thick gingival phenotypes was related to the gingival landmarks at the different apico-coronal levels of assessment with respect to GM (1 and 2 mm apical to the gingival margin) and BM (1, 2, and 3 mm apical to ABC) [20]. This was in contrast to other investigations suggesting consistent GT and BT regardless of the assessment level [12]. The primary distinction between these results was the selected landmarks (gingival margin or ABC) and the depth of their apico-coronal levels. Notably, our study used the ABC as the selected landmarks and explored up to 4 mm apical to the ABC. Consequently, we observed that characteristics like taller labial SGH, wider crowns, flatter gingival margins, higher interdental PH, thicker labial GT, and BT were predominantly identified within 2 mm apical from the ABC. In terms of GM, our results matched those of Arora, who observed a strong positive correlation between the GT and the SGH [14]. Conversely, Ramírez et al. found smaller SGH in thick biotypes compared to mixed and thin periodontium [39], while Fischer demonstrated no significant link between GT and SGH. Concerning BM, it was observed that shorter SGH corresponded with thicker BT [17]. A potential explanation for this difference could be attributed to the assessment techniques used and the specific population selected for measuring the thickness of both the gingiva and the alveolar bone. Previous literature has noted that individuals with broader tooth crowns, abbreviated papillae, flatter gingival angles, and thicker gingiva were less susceptible to gingival recession [5, 40, 41]. This observation may support the hypothesis that individuals with higher SGH could contribute to enhanced resistance to periodontal attacks and increased resilience against the risk of gingival recession. CEJ-ABC is a significant factor that can contribute to periodontal attachment loss and margin recession [15, 42, 43]. This study indicated that a negative correlation between CEJ-ABC exhibited thicker GT and BT on the facial. This result was in accordance with earlier observation reported by Silva and colleagues, which showed found that taller CEJ-ABC measurements were associated with thinner GT and BT [24]. Notably, a higher CEJ-ABC heightens the risk of gingival recession. Consequently, for anterior implant restorations in individuals with elevated CEJ-ABC, it might be prudent to moderately overcompensate during soft tissue augmentation procedures.

Additionally, SGH and CEJ-ABC on the buccal site displayed higher mean values than palatal SGH-p and CEJ-ABC-p, which was consistent with previous studies [12, 14]. On the palatal side, a negative correlation was observed: taller SGH and smaller CEJ-ABC corresponded with thicker gingiva at several sites, specifically at 2 and 4 mm apical to the ABC. To date, previous studies on the palatal PP, especially in the anterior aesthetic region, have been limited. The limited attention might be attributed to the less obvious aesthetic role of the palatal site. However, a comprehensive understanding of PP is imperative in modern aesthetic restorations. In the context of the biologically oriented preparation technique (BOPT), both labial and palatal periodontal tissues should be considered to avoid infringing on the biological width. Such infringements could lead to gingival inflammation and subsequent restoration failure. Therefore, deepening our knowledge of the palatal periodontal characteristics is crucial.

Another important finding was that considering angular metric, SGH exhibited a significant positive correlation with GA and PA, while there was a negative correlation with PH on the buccal side. Conversely, there was also a significant negative correlation between CEJ-ABC and GA, PA, BMA, and IBA. This finding matched those observed in earlier studies where the GA of a thin biotype appeared smaller, and its gingival margin was more curved compared to its thicker counterpart [28, 44, 45]. Various investigations demonstrated that a thicker gingival phenotype often correlated with wider tooth crowns and more robust, shorter gingival papilla [46–50]. The contour of gingival margin is primarily influenced by parameters like GA, PW, and PH [45]. Therefore, when designing the morphology of anterior implant restorations, SGTDs was a critical factor in guiding the aesthetic contour of the gingival margin. In terms of soft tissue angles (GA, BMA, PA, and IBA), there was a positive correlation with hard tissue counterparts (PH and IBH). Thus, we speculated that it was reasonable to postulate that future investigations could perceive periodontal tissues and the underlying bone as an integrated gingiva-osseous complex, collaboratively fortifying against periodontal adversities. Regarding IBH, Becker and colleagues conducted a comprehensive analysis of alveolar bone anatomical profiles using dry skulls, providing valuable insights into alveolar bone structure [51]. They reported IBH based on bone anatomic morphotypes (flat: 2.1±0.51 mm; scallop: 2.8±0.56 mm; and pronounced scalloped: 4.1±0.60 mm). In contrast, our study observed IBH values of 2.63 ± 0.48 mm, indicating a slight difference compared to the measurement reported by Becker et al. This disparity can be attributed to both differences in the studied populations and variations in research methodologies. Furthermore, tooth type exhibited a strong correlation with SGH and CEJ-ABC on either the buccal or palatal side. Central incisors exhibited the highest mean values of SGH (3.46 ± 0.58 mm) whereas canines presented the highest CEJ-ABC-p (1.06 ± 0.30 mm) and SGH-p (3.21 ± 0.54 mm). This result was in contrast with previous studies that reported no statistical significance in the median values of SGH between central incisors (3.00 ± 1.20 mm), lateral incisors (3.00 ± 0.90 mm) and canines (3.00 ± 1.00 mm) on the buccal and palatal sites [14]. This inconsistency may be attributed to the chosen statistical value and measurement methods.

Despite the meticulous design of the experiment, there are still some limitations to this study. To begin with, our focus was solely on the maxillary anterior teeth. As a result, there is a need for future research to include other teeth types like premolars, molars, and those in the mandibular arch. Additionally, the interproximal SGTDs were omitted from our study. Another point to consider is that our study subjects were aged between 18 and 25 and of Han nationality. Hence, it was unsure whether our results were applicable to other groups, raising questions about the applicability of our findings to broader populations. It would be valuable for future studies to encompass middle-aged and elderly participants to evaluate age-dependent variations in periodontal biotype. Lastly, our results hinted at the significance of facial data. Recognizing that aesthetic design should incorporate information on the dental arch, jawbone, and facial metrics, our research intends to further explore the association between facial metrics and PP using integrated digital methods that include CBCT, intraoral scanning and facial scanning techniques for the most harmonious aesthetic outcome.

## Conclusion

Within the limitation of this cross-sectional study, the conclusions were drawn as follows:

1. SGTDs exhibited a correlation with other PP components, especially crown shape, gingival margin and interdental PH.
2. The relationship between SGTDs and gingival and bone phenotypes depended on the apico-coronal level evaluated.

### Electronic supplementary material

Below is the link to the electronic supplementary material.

Supplementary Material 1

## Data Availability

The datasets generated and/or analysed during the current study are not publicly available due the policies and confidentiality agreements adhered to in our laboratory but are available from the corresponding author on reasonable request.

## Abbreviations

SGTDs: Supracrestal gingival tissue dimensions
PP: Periodontal phenotype
ABC: the crest of alveolar ridge
CEJ: Cementoenamel junction
SGH: Supracrestal gingival height
CEJ-ABC: The distance from the cementoenamel junction to the crest of the alveolar ridge
CBCT: Cone beam computed tomography
IOS: intraoral scanning
CM: Crown morphology
GM: Gingival morphology
BM: Bone morphology
IOS: Intraoral scanning
GT: Gingival thickness
BT: Bone thickness
CL: Crown length
CW: Crown width
CW/CL: Crown width/crown length ratio
GA: Gingival angle
PA: Papilla angle
PW: Papilla width
PH: Papilla height
DICOM: Digital imaging and communication in medicine
BMA: Bone margin angle
IBA: Interproximal bone angle
IBH: Interproximal bone height
SGH-p: Palatal supracrestal gingival height
CEJ-ABC-p: Palatal distance from cementum-enamel junction to the crest of alveolar ridge
GT_cej_: Facial gingival thickness at the cementum-enamel junction
GT_cej_-p: Palatal gingival thickness at the cementum-enamel junction
GT_abc_: Facial gingival thickness at the crest of the alveolar ridge
GT_abc_-p: Palatal gingival thickness at the crest of the alveolar ridge
GT_1_: Facial gingival thickness to at 2 mm apical the crest of the alveolar ridge
GT_1_-p: Palatal gingival thickness to at 2 mm apical the crest of the alveolar ridge
GT_2_: Facial gingival thickness to at 4 mm apical the crest of the alveolar ridge
GT_2_-p: Palatal gingival thickness to at 4 mm apical the crest of the alveolar ridge
BT_1_: Facial bone thickness to at 2 mm apical the crest of the alveolar ridge
BT_1_-p: Palatal bone thickness to at 2 mm apical the crest of the alveolar ridge
BT_2_: Facial bone thickness to at 4 mm apical the crest of the alveolar ridge
BT_2_-p: Palatal bone thickness to at 4 mm apical the crest of the alveolar ridge
ICC: The intraclass correlation coefficient
ANOVA: One-way analysis of variance
