# Supplementary material for: Assessment of the correlation between supracrestal gingival tissue dimensions and other periodontal phenotypes components via the digital registration method: a cross‑sectional study in a Chinese population
Source: BMC Oral Health. 2024 Apr 1;24:408. doi: 10.1186/s12903-024-04158-0 (PMC10985880; doi:10.1186/s12903-024-04158-0)
Supplement: Supplementary file 1 — Supplementary Material 1 [file 12903_2024_4158_MOESM1_ESM.docx]

**Participants were excluded (N=51)**

**8 participants were excluded due to a history of smoking, previous orthodontic treatment, or root canal treatment**

**43 participants were excluded due to crowding or misalignment of the maxillary anterior**

**Initial screened**

**(N=134)**

**Participants fulfilling the inclusion criteria (N=83)**

**Intraoral scanning procedure**

**Radiography examination procedure**

**Reconstruction and measurement of digital models**

**Statistical analysis**

**Fig 1. Flow chart of the trial protocol**
